# Supplementary figures and images for: Genomic analysis on broiler-associated Clostridium perfringens strains and exploratory caecal microbiome investigation reveals key factors linked to poultry necrotic enteritis
Source: Anim Microbiome. 2019 Oct 18;1:12. doi: 10.1186/s42523-019-0015-1 (PMC7000242; doi:10.1186/s42523-019-0015-1)

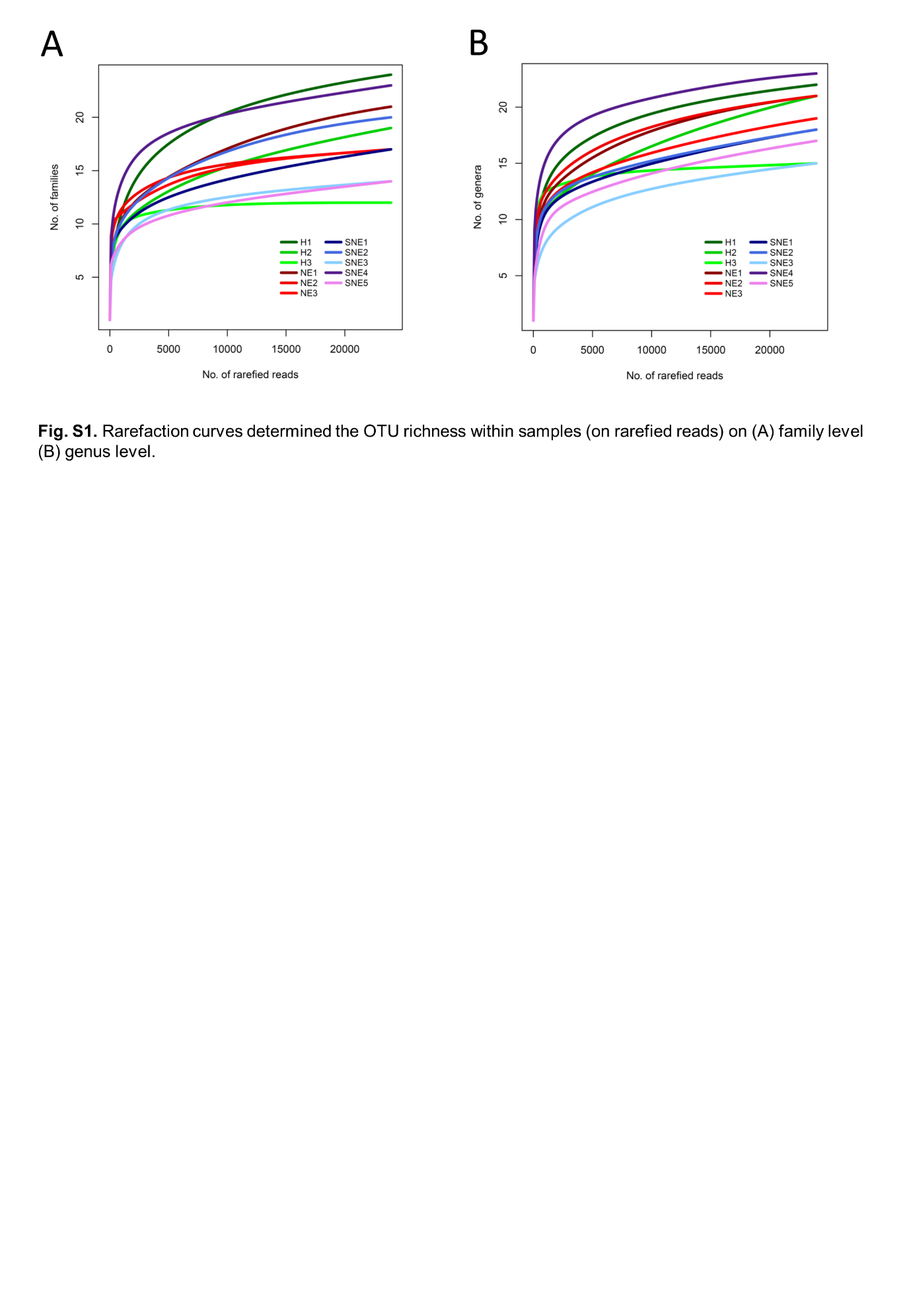

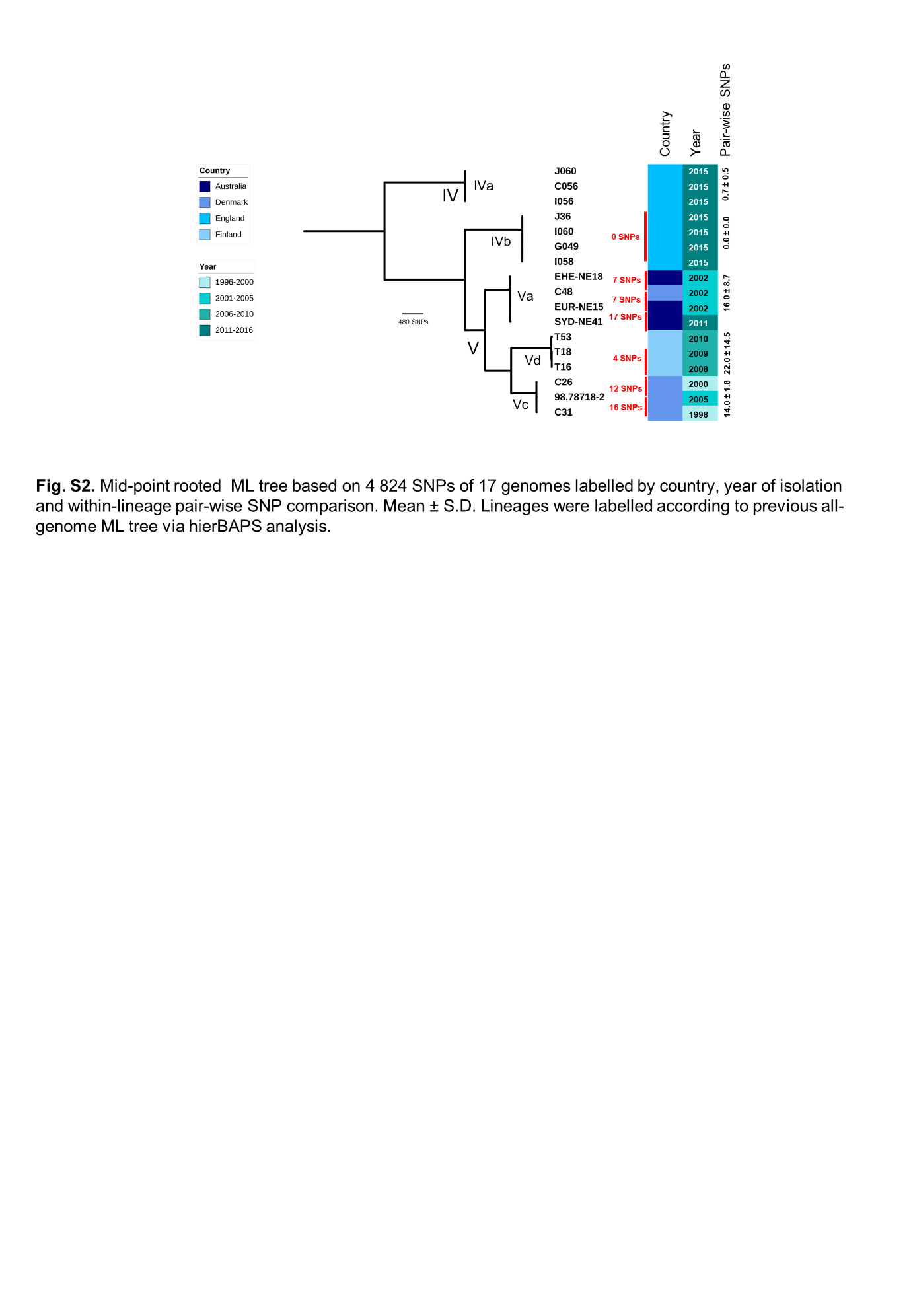


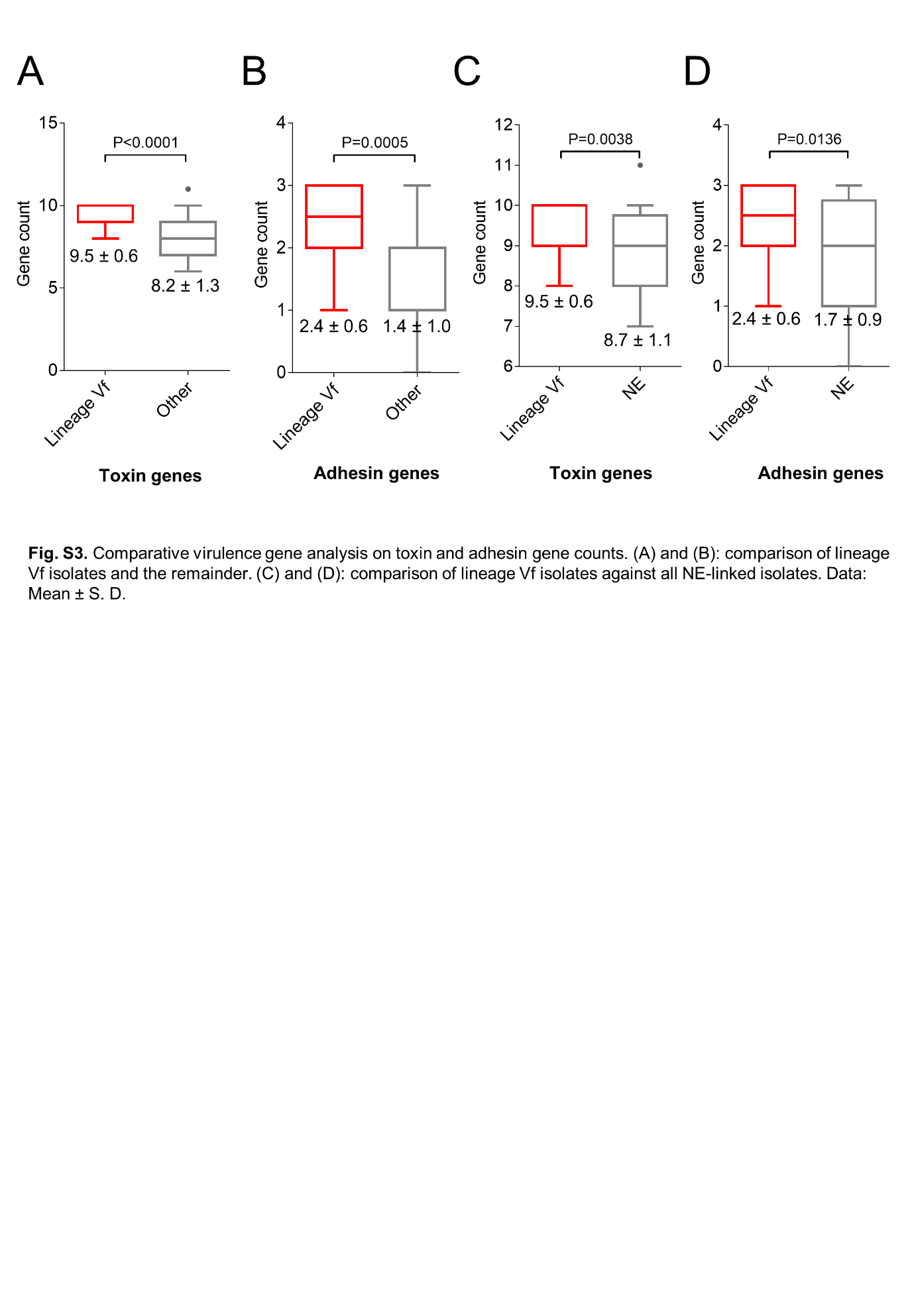


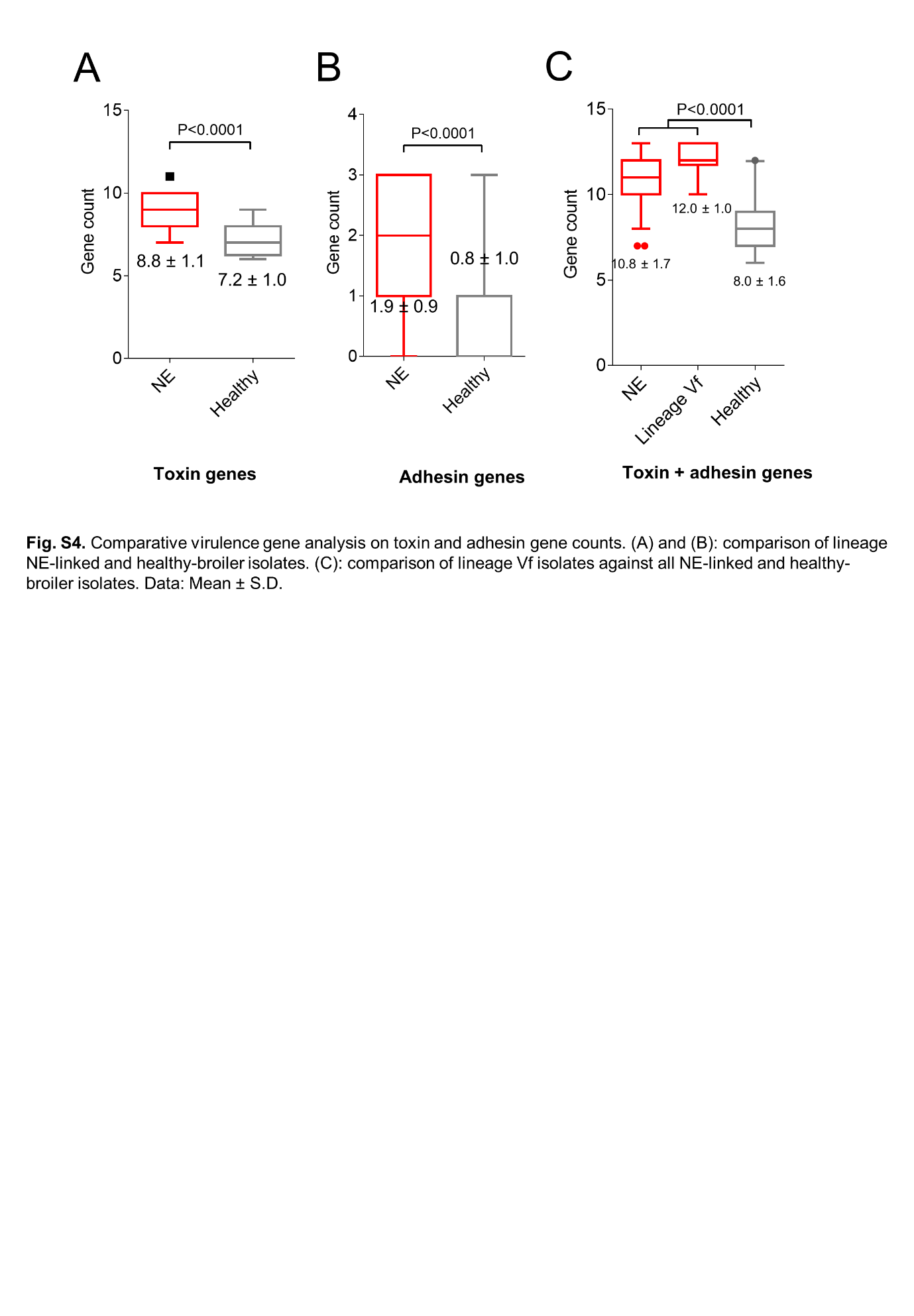


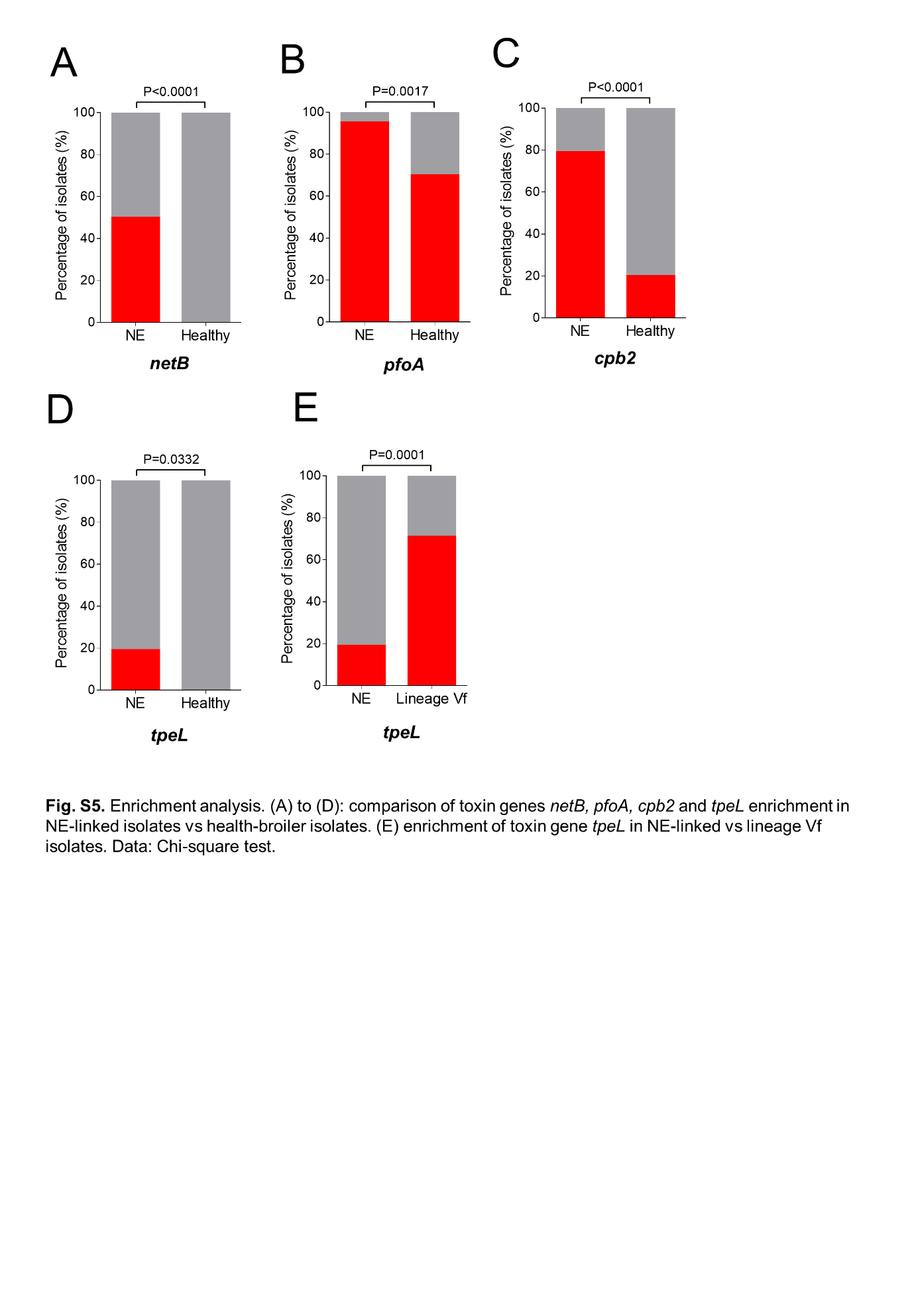


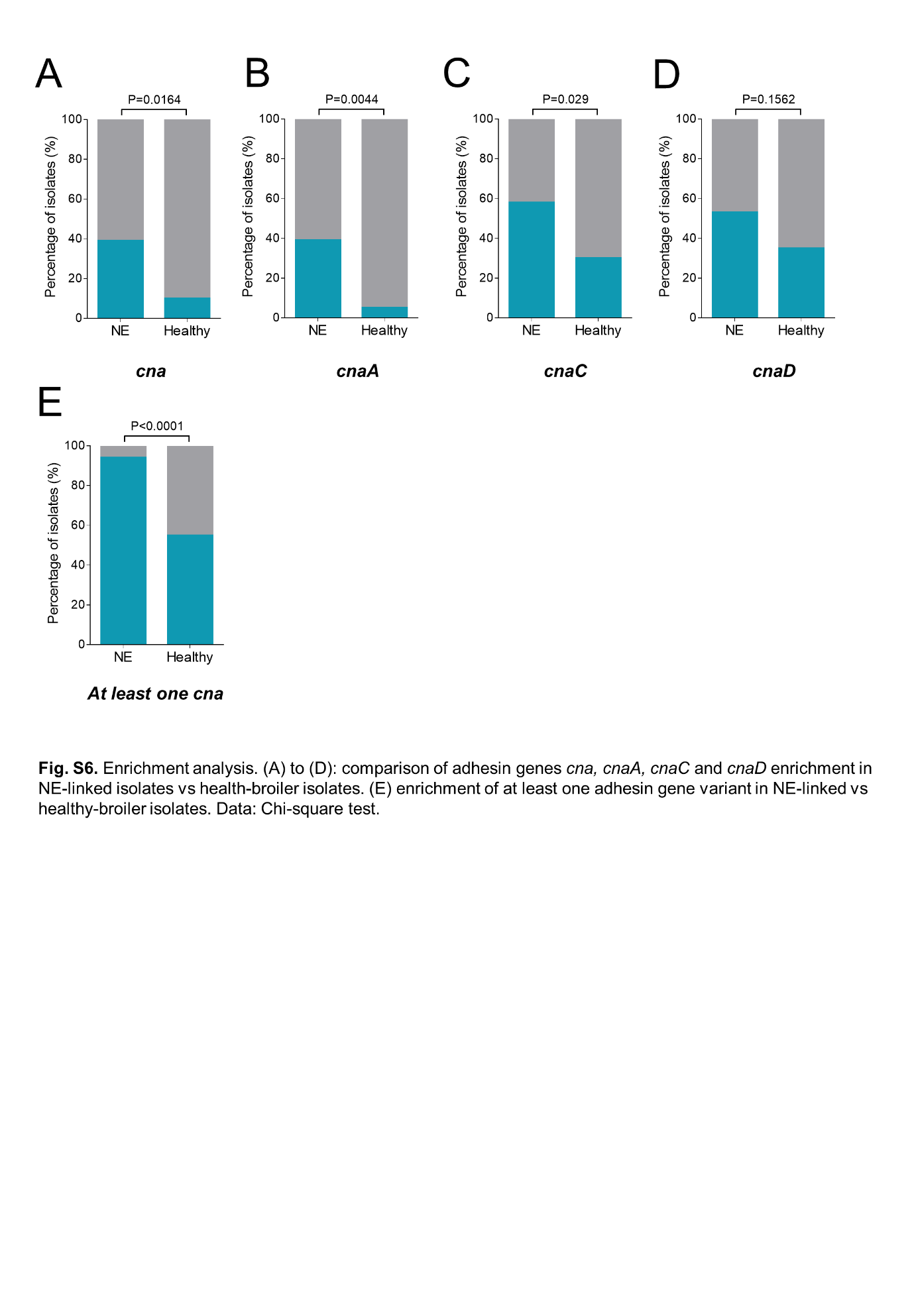


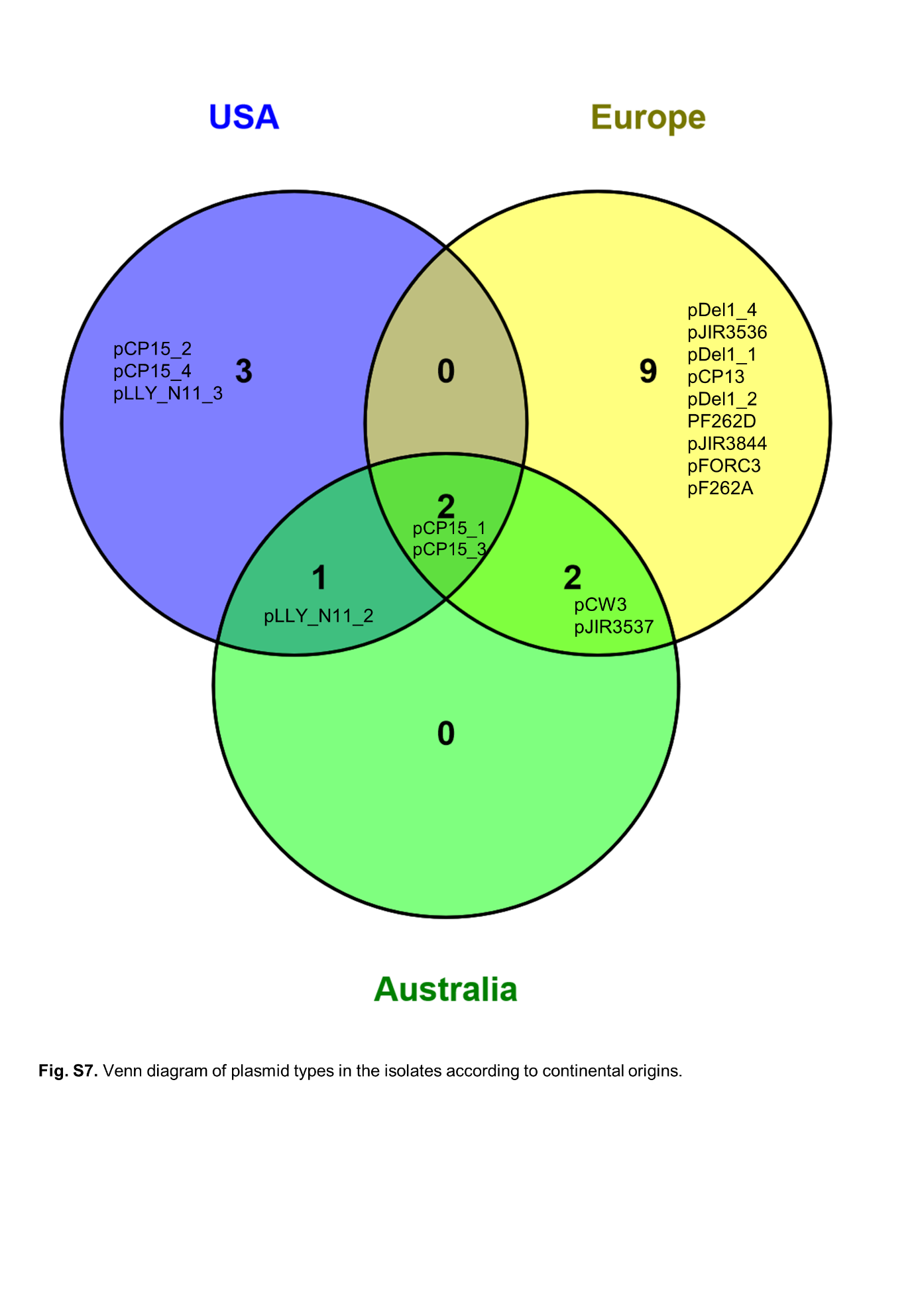


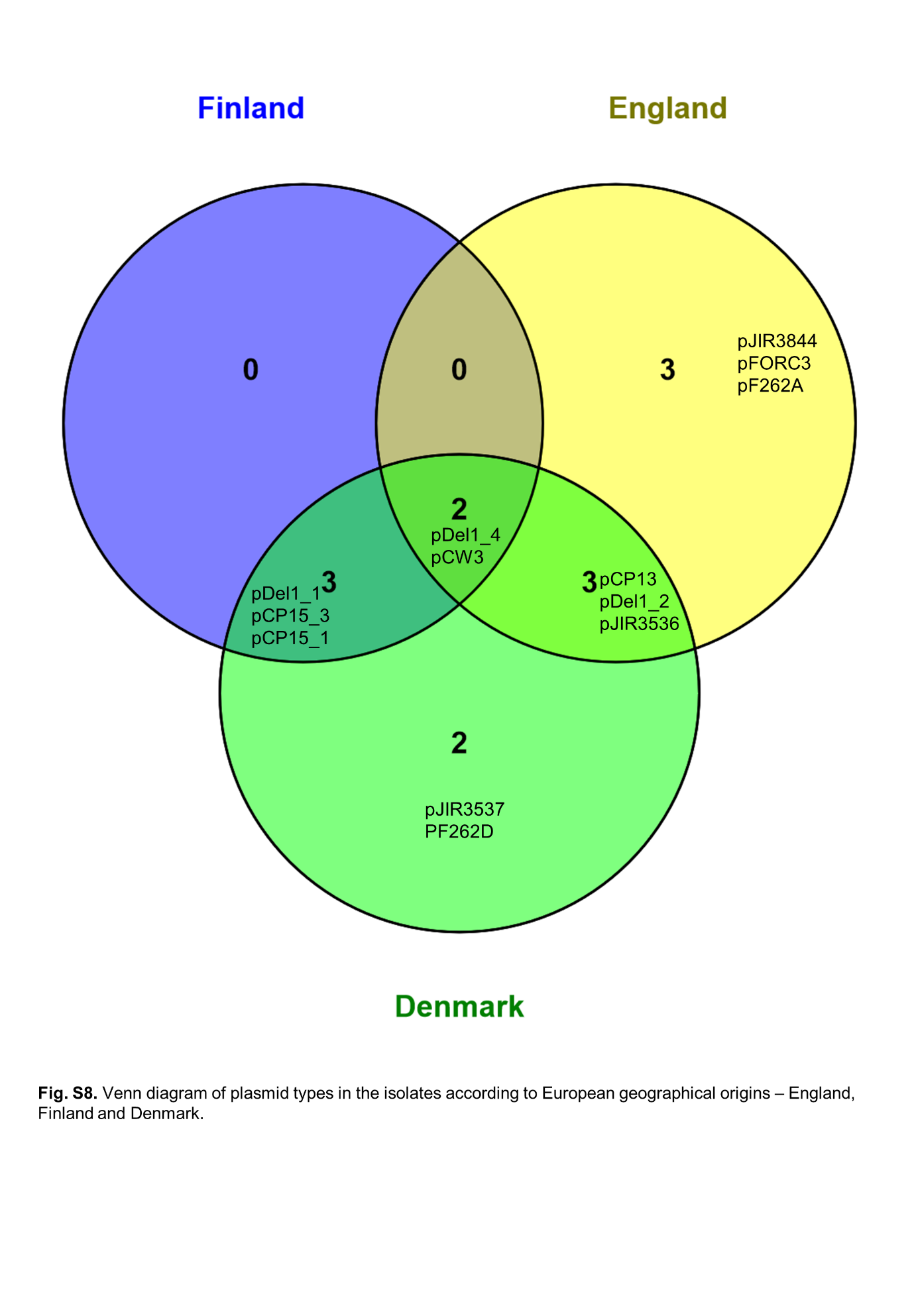


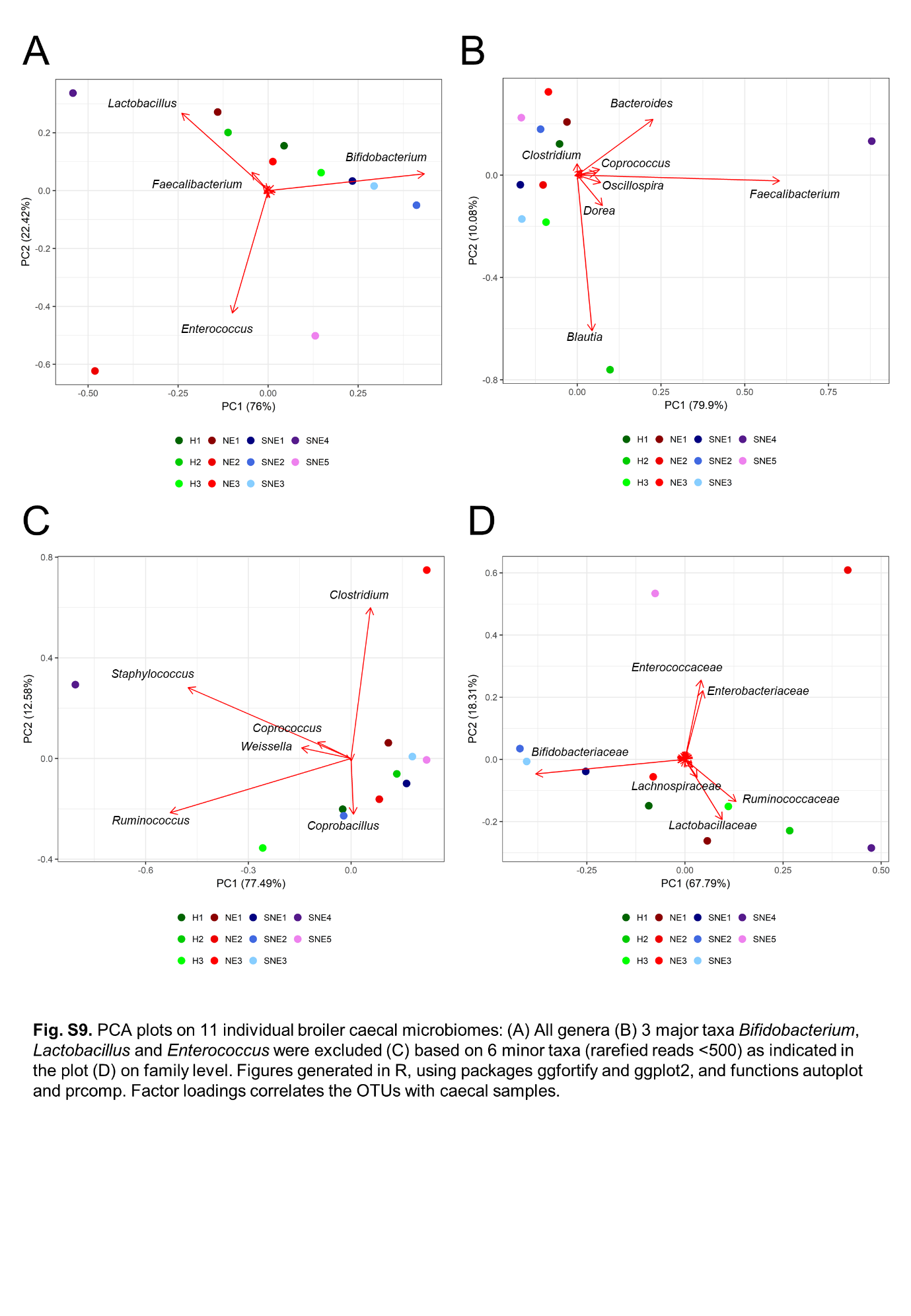

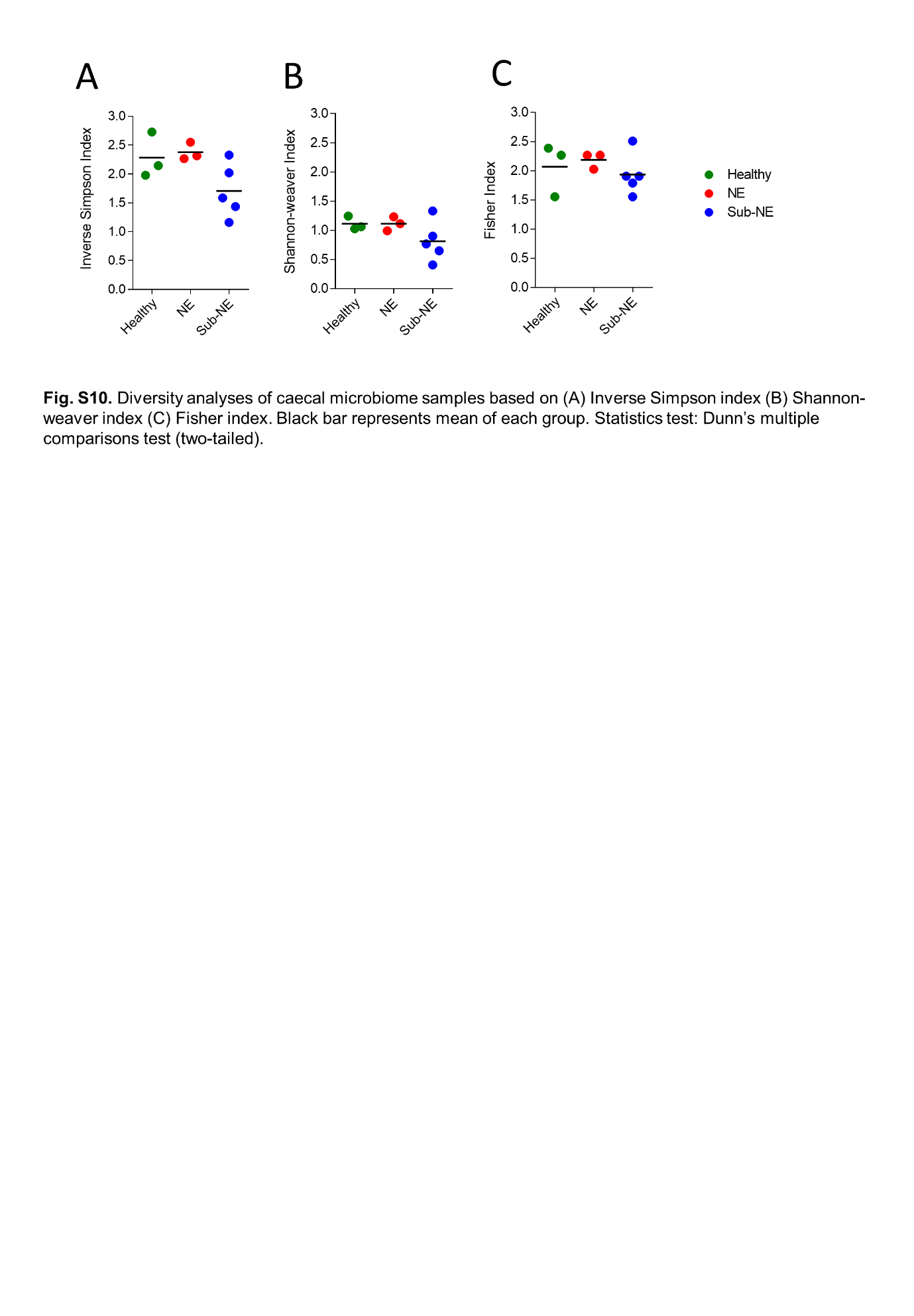


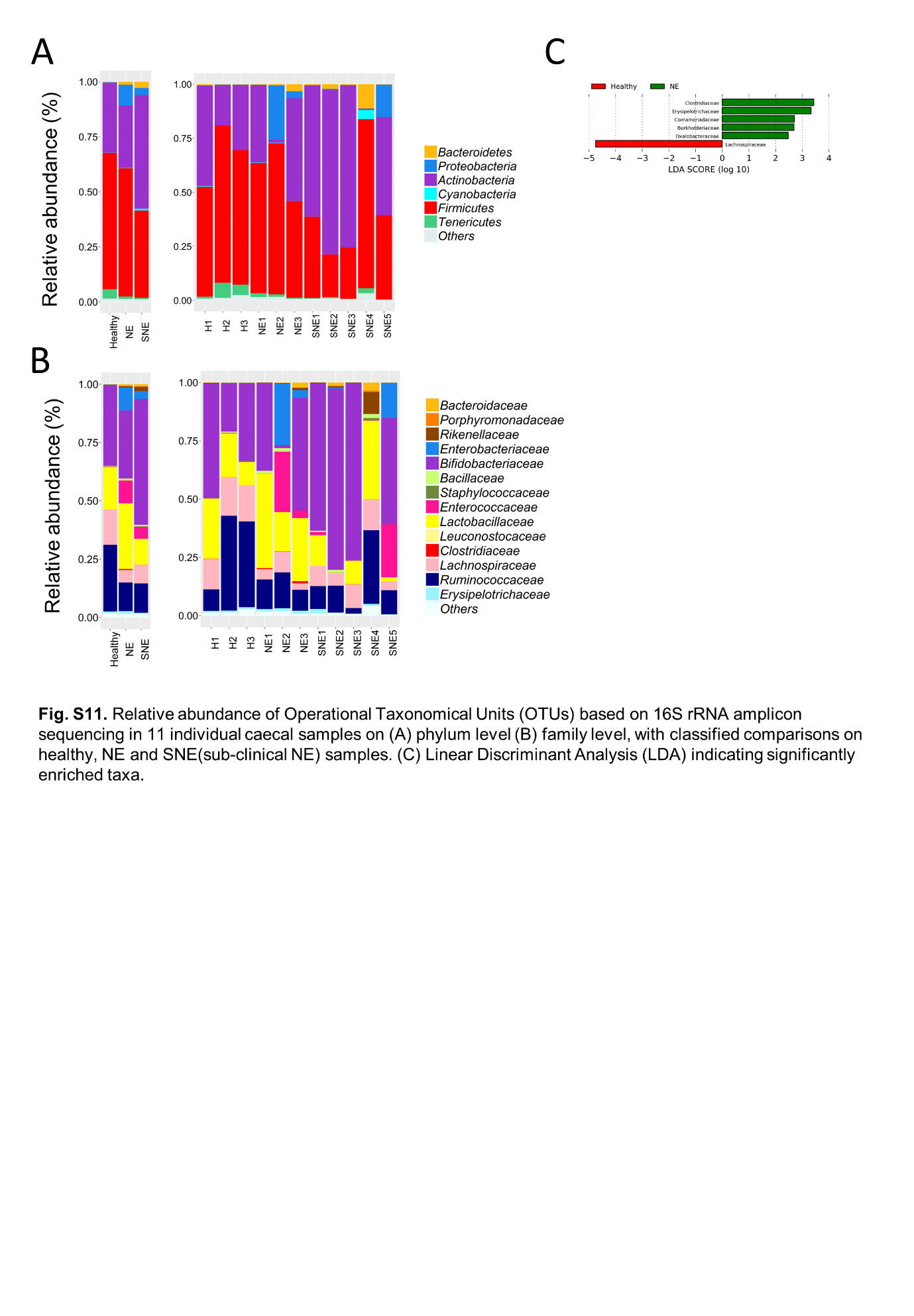


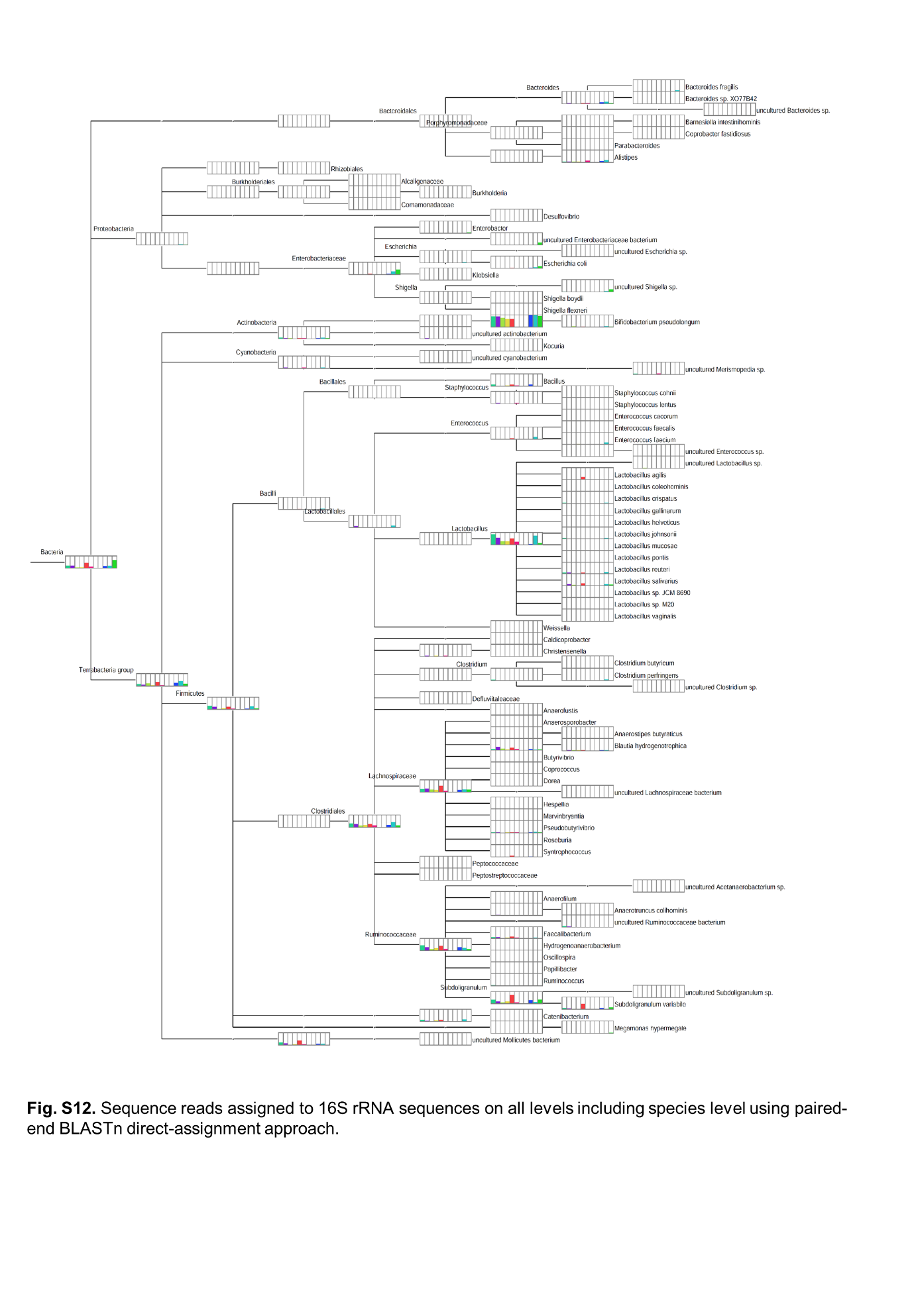

Supplement: Supplementary file 2 — Additional file 2. Supplementary figures. [file 42523_2019_15_MOESM2_ESM.docx]
